# Influence of Person-Vocation Fit on Satisfaction and Persistence in Vocational Training Programs

**DOI:** 10.3389/fpsyg.2022.834543

**Published:** 2022-02-14

**Authors:** Christian Michaelis, Stefanie Findeisen

**Affiliations:** ^1^Chair of Business Education and Human Resource Development, Faculty of Business Economics, Georg-August University of Göttingen, Göttingen, Germany; ^2^Assistant Professorship for Business and Economic Education, Department of Economics, University of Konstanz, Konstanz, Germany

**Keywords:** person-vocation fit, interest congruence, job satisfaction, premature contract termination, transition from school to work, vocational education and training (VET)

## Abstract

Positive effects of person-environment fit on job satisfaction and persistence are well documented. However, little is known about the consequences of person-vocation (P-V) fit for vocational education and training (VET). Using data from the German National Educational Panel Study (NEPS), we examine the influence of selected P-V fit indicators (educational match, interest congruence, skill congruence) on training satisfaction and premature contract termination (PCT) for 4,097 trainees in VET. We find that most P-V incongruences do not lead to negative consequences. Training satisfaction is not affected by interest congruence and skill congruence. However, moderate overeducation (intermediately qualified adolescents working in occupations with high shares of low-qualified trainees) enhances training satisfaction. For PCT, there is a general effect of undereducation; undereducation increases the probability of PCT independent of educational qualification for the occupation. PCT is not affected by skill congruence and only for Realistic interests, congruence with the vocational environment reduces PCT probability.

## Introduction

At the end of formal education, adolescents face the task of choosing an occupation and hence make an important and far-reaching decision about their future. This decision has consequences for their future career success (e.g., range of jobs available to individuals, future development and achievement) (Savickas, 1999). In many countries (e.g., Austria, Denmark, Germany, United Kingdom, Switzerland), the way to start a career—apart from higher education—is a vocational education and training (VET) program in cooperation with a company (Organisation for Economic Co-operation and Development [OECD], 2010, p. 9; Poulsen and Eberhardt, 2016, p. 6). In Germany, which is the focus of our study, VET programs have a strong tradition. The number of adolescents participating in formal education is equally divided into VET and general education for the age group of 16–19 and between VET and higher education for the age group of 19–25 (Autorengruppe Bildungsberichterstattung, 2020, p. 64). In this paper, we will focus on the largest sector of German VET, the dual system (324 occupations, Bundesinstitut für Berufsbildung [BIBB], 2020, p. 324; beginning trainees in 2019: 492.276, Autorengruppe Bildungsberichterstattung, 2020, p. 152; for further explanations on the German VET system, see Solga et al., 2014). Here, trainees are employed by a company where they are included in the daily work in their profession and instructed on practical skills by a trainer. They also attend vocational schools—typically one to two days per week—where they acquire theoretical knowledge relevant for their profession. There are no formal qualification requirements to start a dual vocational training program, as companies decide whom to hire. However, the majority of trainees in dual vocational training programs has an intermediate school-leaving qualification (Seeber et al., 2019a). Consequently, VET programs are especially important for this group of adolescents who do not fulfill qualification demands of higher education (Holtmann et al., 2017, p. 2092), but VET programs are also an important alternative to higher education programs for adolescents with university entrance qualification.

Attending a VET program is, however, not just about acquiring skills; adolescents also become socialized to the world of work (Heinz, 2002; p. 238). Thus, the successful completion of a VET program is a central requirement for career success during individuals’ working life (European Centre for the Development of Vocational Training [CEDEFOP], 2013, p. 56). However, several studies reveal that the transition from school to VET is a challenging process, because adolescents’ needs and training providers’ demands and thus training claims have to be well matched to prevent frictions in the training process (Michaelis and Busse, 2021, p. 91 f.; Protsch and Solga, 2015; Forsblom et al., 2016). Especially in the case of a much differentiated occupational VET system (like Germany, Switzerland, Austria) which often competes with additional educational alternatives, adolescents have to choose between several alternatives. Evidence shows that adolescents’ career aspirations are not always realistic (Musset and Kurekova, 2018; Hoff et al., 2021;, p. 30) and the decision for a specific occupation is often not appropriate in comparison to adolescents’ skills. In the context of dual VET programs, the responsibility for filling training positions adequately lies on the side of training providers. However, selecting suitable trainees is also challenging for training providers due to the nature of the applicant pool (number of applicants, quality of applications), asymmetries of information about applicants, competition with other training providers and legal requirements for filling positions that can limit selection criteria (Michaelis and Busse, 2021, p. 91 f.; Dummert et al., 2019). Thus, there are multiple barriers to fill training positions optimally, regularly leading to mismatches regarding trainees and their training occupation.

The consequences of such mismatches depend in particular on the effectiveness of work-adjustment processes, during which individuals strive to achieve and maintain congruence with the work environment (Dawis and Lofquist, 1984). If an individual’s needs are fulfilled by the occupational environment, he or she will be more satisfied with work. If incongruences interfere with performance in the workplace, the probability of frictions in the work process increases. These frictions can foster dissatisfaction with the job and negatively affect job persistence. Hence, person-environment (P-E) fit is an important indicator of career success. In career research, it has been well documented that both job satisfaction (e.g., Hardin and Donaldson, 2014; Gander et al., 2020) and persistence intention are positively affected by P-E fit (e.g., Kristof-Brown et al., 2005).

The VET sector is regularly confronted with high rates of premature contract terminations (PCTs), which are reported for VET programs across countries (e.g., Austria: 16.9%, Dornmayr and Nowak, 2019, p. 65; Germany: 26.5%, Bundesinstitut für Berufsbildung [BIBB], 2020, p. 11; Switzerland: 26%, Bundesamt für Statistik, 2019, p. 5). While PCTs can be both the cause and a means of adjusting occupational choices, they have costs for all parties involved in VET. For individuals, PCTs are likely to be connected to an experience of failure and can---for instance, if not initiated by the trainee^1^ —lead to uncertain conditions, a search for alternatives and, at worst, unemployment. Additionally, for companies investing in VET programs as well as from a societal perspective, PCTs reflect an inefficient allocation of resources. Hence, it seems worthwhile to examine to what extend P-E fit in VET can explain PCT.

Compared to the well-documented positive effect of P-E fit on satisfaction and persistence in career research, evidence for the VET context is still scarce. Existing research does, however, hint toward a positive effect of a perceived person-vocation (P-V) fit (one form of P-E fit) on training satisfaction as well as on the intention to complete the training program (Nägele and Neuenschwander, 2015, p. 67). The present study aims to examine whether the relationship between indicators of P-V fit (educational match, vocational interest congruence, vocational skill congruence) and satisfaction as well as persistence also holds for the context of VET and to what extend fit indicators contribute to the high rates of PCT.

## Person-Vocation Fit

### Conceptualizing Person-Vocation Fit

Person-environment fit refers to the congruence between an individual and his or her work environment. Congruence can occur on different levels, e.g., the job, the organization, the work group or the vocation (Kristof-Brown et al., 2005). The broadest of these levels is the vocation. Person-vocation (P-V) fit refers to “the congruence between individuals’ interests and abilities and the characteristics and requirements of their vocation” (Vogel and Feldman, 2009, p. 70). Although there is some overlap between P-V fit and person-job fit (P-J fit), the two concepts differ with regard to the reference point of fit. While P-V fit addresses the congruence of skills and interests for a certain vocation, P-J fit refers to a specific position. Within the broader literature of P-E fit, P-V fit has been less frequently studied. However, P-V fit is regarded as an important antecedent of person-organization fit and person-job fit, as persons with low P-V fit will be less successful in their careers regardless of the specific job and organization they work in Vogel and Feldman (2009). We claim that P-V fit is especially important when analyzing the transition from school to VET, as it is related to career counseling, the process of vocational choice as well as work adjustment. Hence, the concept is rooted in theories of both vocational choices and work adjustment. Vocational choice theories (e.g., Holland, 1997) propose that individuals choose careers in line with their interests and their self-concepts. According to the choice model of the Social Cognitive Career Theory (SCCT) interests are expected to be the most important determinant of career decisions (Lent and Brown, 1996). Empirical evidence provides support for this assumption (e.g., Volodina and Nagy, 2016). According to Holland (1997), the congruence between an individual’s personality and his or her work environment is expected to affect both satisfaction and stability. SCCT also links vocational interests to satisfaction and persistence intentions (e.g., Lent et al., 2015).

### Early Career Stage Person-Vocation Fit Indicators and Their Effects on Satisfaction and Persistence

**FIGURE 1:**
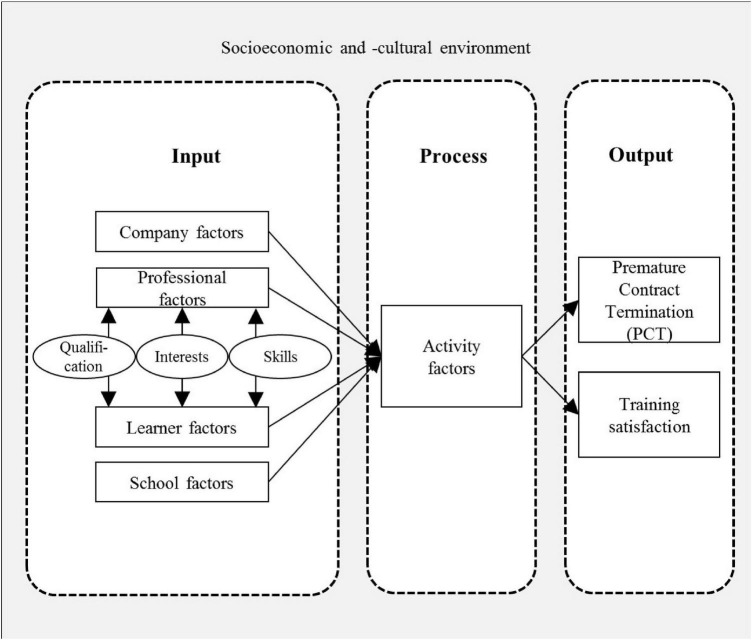
3-P model: Reasons for PCT and training satisfaction (adapted from Böhn and Deutscher, 2022; modified from Tynjälä, 2013).

Figure 1 displays an adapted version of the 3-P model explaining output variables in VET (Böhn and Deutscher, 2022; modified from Tynjälä, 2013). The model shows that PCT and training satisfaction (as the output variables that are examined in this study) are influenced by both input factors and process factors. Apart from company factors and factors of the vocational schools, prerequisites of trainees (e.g., education, age, gender, motivation) as well as professional factors (e.g., career choice, professional identity) are relevant presage conditions for the training process and, hence, for output variables.

In our study, we are especially interested in the effects of P-V fit. While P-V fit was not explicitly included in the original version of the model in Figure 1, it is highly plausible that the congruence between individual factors and professional factors also affects the training process and output. Regarding indicators of P-V fit, we concentrate on those vocational demands that differ between occupations and person-related constructs that have the development potential to enhance the professionalism of an individual in the early career stage. Research on transitions from school to VET reveals that school-leaving certificates are an important factor when it comes to obtaining a training position (Protsch and Solga, 2015; Holtmann et al., 2017; Michaelis and Busse, 2021). Therefore, a first important P-V fit indicator—for VET systems where access conditions to training positions are less restrictive (e.g., in Germany) —is educational match. Since an analysis of educational match does not allow for a differentiated view on domain- and occupation-specific demands, we use two additional indicators of P-V fit to measure a fit between occupation-specific requirements and individual characteristics. In detail, we examine interest congruence based on the RIASEC model (Holland, 1997) as well as vocational skill congruence.

#### Educational Match

A school-leaving certificate is an objective source of information about previous school achievement and demonstrates a specific level of educational qualification. On a high aggregation level this reflects the skills acquired during previous education. The educational qualification is seen as an indicator of adolescents’ trainability and thus their ability to cope with training demands for training providers in the selection process (Michaelis and Busse, 2021, p. 92 f.; Protsch and Solga, 2015, p. 525). Empirical findings reveal that training occupations in the German training market differ strongly by educational qualification levels of beginning trainees (Autorengruppe Bildungsberichterstattung, 2016, p. 286) which indicates that for different professions, different levels of educational qualification are expected. In this context, educational match refers to “a situation in which the level of education attained by an individual does (not) correspond to the educational requirements of her job” (Mateos-Romero and Salinas-Jiménez, 2018, p. 370). As a consequence, employees can experience both under- and overeducation. Most empirical investigations have found that only overeducation has a significant effect on employees’ work attitude, and it decreases job satisfaction (Sánchez-Sánchez and McGuinness, 2015, p. 425; Verhaest and Verhofstadt, 2016). The reason is a limited realization of an individual’s abilities and skills. The educational match of a training relationship must be evaluated in relation to the school-leaving certificates expected before the start of training. There are no known studies that analyze the effects of educational match on training satisfaction. However, general findings of overeducation show that dissatisfaction exists mainly in the early career stage (Verhaest and Verhofstadt, 2016). Hence, such effects can be expected in the VET context as well. To explain PCT in VET programs by educational match, Rohrbach-Schmidt and Uhly (2015, p. 122 f.) show that under- and overeducation among German trainees have hardly any additional explanatory power to explain PCT (only weak effects) if the educational level of trainees is controlled for. However, their results document that low education represents one of the highest risk factors for PCT (see also Beicht and Ulrich, 2009; Bundesinstitut für Berufsbildung [BIBB], 2020, p. 30). The findings of Rohrbach-Schmidt and Uhly (2015, p. 128 f.) show that the disadvantages of trainees with low education are not substantially reinforced by occupations characterized by higher requirements but are evident across occupations.

#### Vocational Interest Congruence

In the RIASEC model (Holland, 1997), fit relates to the congruence between individual vocational interests and characteristics of the occupational environment (vocational interest congruence). Theoretically, people prefer a working environment that matches their affinity. In this regard, interests have a motivational force. Interest congruence can intensify employees’ commitment, increase their job satisfaction, lead to persistence at work, and improve job performance (Lent and Brown, 1996; Holland, 1997). These effects have been documented in several studies (van Iddekinge et al., 2011; Hoff et al., 2020).

For the assessment of vocational interests the RIASEC model by Holland (1997) distinguishes six dimensions: (R) Realistic, (I) Investigative, (A) Artistic, (S) Social, (E) Enterprising, and (C) Conventional. Each occupation and person can be individually characterized and congruence can be modeled by a comparison of individual dispositions and job characteristics. As not every RIASEC dimension is equally important for an occupation, it has become common practice to limit the analysis to the dominant RIASEC dimensions (Ertl and Hartmann, 2019). Recent meta-analyses reveal the importance of interest congruence in P-E fit research (Hoff et al., 2020). Some studies have shown that age has a moderating effect and indicated that the RIASEC model is valid for younger groups (Meir, 1999; Tsabari et al., 2005), since vocational interests are of higher importance in early career stages. With increasing age, other factors become decisive explaining job satisfaction (“adjustment, skill utilization, burnout, experience, advancement opportunities, compensation, status, social acceptance;” Tsabari et al., 2005, p. 220). In addition, Hoff et al. (2022) show that interest congruence in the early career stage has long term effects as it increases job and career satisfaction as well as occupational prestige in later employment. Furthermore, a meta-analysis by van Iddekinge et al. (2011) shows that interests (measured based on the RIASEC model) predict training performance.

To date, there are hardly any findings regarding an indirect, objectively measured P-V fit and its consequences for the training process. However, Volodina et al. (2015) illustrate that individual dimensions of vocational interests (based on the six RIASEC dimensions) predict training satisfaction and intention to terminate the training contract prematurely for industrial clerks and technical trainees. In particular, Realistic interests seem to be predictive of persistence in VET for technical trainees and of leaving VET for industrial clerks. Additionally, Etzel and Nagy (2021) show that vocational interest congruence is important for training satisfaction also for industrial clerks and technical trainees. However, these studies cover only a small range of occupations, so no direct conclusions can be drawn about the influence of interest congruence on the training process. More importantly, only *intentions* to terminate the training contract and no actual PCTs are examined by Volodina et al. (2015).

#### Vocational Skill Congruence

Vocational education and training programs prepare trainees for future requirements and activities of a specific occupation. Therefore, it is important to acquire occupation specific skills in order to cope with demands in an occupation. According to Winther and Achtenhagen (2009, p. 92), *domain-linked* abilities and skills are necessary for development within a specific occupation. These are general and basal occupational key skills like numeracy and literacy, which facilitate solving specific VET problems (Winther and Achtenhagen, 2009, p. 92). Empirical findings show differences in *domain-linked* abilities and skills of beginning trainees in occupational group specific analyses (Lehman and Hunger, 2007; Autorengruppe Bildungsberichterstattung, 2018;, p. 33ff), which indicate different levels of expected *domain-linked* abilities and skills in VET. Furthermore, several studies show that skills such as mathematical and scientific competences or language skills are important explanatory predictors of training success (Rosendahl and Straka, 2011, p. 26; Seeber and Lehmann, 2013, p. 79 f.). In a previous study based on selected occupations, Volodina et al. (2015) could, however, not confirm any influence of competence in mathematics or physics on training satisfaction or dropout intention for industrial clerks and technical trainees. This finding is based on a small range of occupations only and cannot be generalized for a broad range of occupations in VET systems. Therefore, in our study, a third indicator of P-V fit deals with the congruence between domain-linked skills and the importance of these skills for the chosen occupation (vocational skill congruence).

## The Present Study

We examine how P-V fit affects (a) training satisfaction and (b) premature contract termination (PCT) in the first year of VET using longitudinal data from the German National Educational Panel Study (NEPS; Blossfeld et al., 2011). The NEPS dataset allows us to thoroughly analyze the complex interactions that play a role during the transition from school to VET. We specifically focus on measures of individual characteristics (interests, skills) before the start of the training for analyzing consequences of P-V fit on the transition process from school to work. A major advantage of our approach is the possibility of analyzing actual PCT. Previous studies (e.g., Volodina et al., 2015) have focused on the intention to terminate a contract. Intention measures are regularly used as indicators for actual termination. However, at least for higher education, studies have shown that dropout intention is only moderately related to actual later dropout (e.g., Lent et al., 2016, p. 84). Our approach allows us to analyze whether the positive relationship between vocational interests and persistence intention found by Volodina et al. (2015) holds true for actual PCT. Another advantage is that our study is not restricted to single occupations but provides implications for a broad range of professions.

We add to prior research on P-E fit in the following ways. While existing research focuses mainly on transitions into working life after completion of a higher education program or training program (e.g., Nordin et al., 2010; Béduwé and Giret, 2011) or on transitions in later working life (Mateos-Romero and Salinas-Jiménez, 2018), we examine whether well-established results from the education-to-work transition can be replicated for the transition from high school to VET. Moreover, our analysis relies on actual fit indices (indirect measures relying on a separate assessment of personal and environmental characteristics), thus avoiding self-evaluation bias concerning P-V fit measures. We use objective information on the environment, which enables a more precise modeling of reality.

We aim to examine the following hypothesis. First, due to theoretical assumptions about the consequences of overeducation (Sánchez-Sánchez and McGuinness, 2015; Verhaest and Verhofstadt, 2016), we expect that overeducation limits self-reported training satisfaction. However, based on the results of Rohrbach-Schmidt and Uhly (2015), we expect to find no significant effects of under- or overeducation on PCT once educational background is controlled for.

H1a: Overeducation decreases training satisfaction when educational background is controlled for; undereducation has no effect on training satisfaction.
H1b: Under- and overeducation do not influence the probability of PCT when educational background is controlled for.

Second, we assume that an incongruence of vocational interests and characteristics of the vocational environment has a negative effect on the training process.

H2a: Training satisfaction increases with increasing interest congruence.
H2b: The probability of PCT decreases with increasing interest congruence.

Third, we expect that lower skill levels in mathematics, reading comprehension and science influence the training process negatively in occupations where these skills are necessary.

H3a: Training satisfaction increases with increasing skill congruence in the domains of mathematics, reading comprehension and science.
H3b: The probability of PCT decreases with increasing skill congruence in the domains of mathematics, reading comprehension, and science.

## Materials and Methods

### Data

We use the SC 4 dataset (version 10.0.0) of the NEPS (Blossfeld et al., 2011; Leibniz-Institut für Bildungsverläufe [LIfBi], 2019). This dataset stems from a longitudinal survey on the development of adolescents in Germany. Since 2010, 16,425 9th grade students have been regularly surveyed on their educational process and career trajectories. The dataset allows analysis of adolescents’ biographies until summer 2017. We reduced the dataset to participants who started a VET program (dual system) after leaving general education (*n* = 5,646). As the NEPS survey is ongoing, the dataset also includes trainees who have not yet completed their training. For our analysis, we selected (1) adolescents whose contract ended prematurely within the first 12 months (where 67% of all PCTs occur, Bundesinstitut für Berufsbildung [BIBB], 2020, p. 143) and (2) trainees whose training period lasted longer than 12 months (*n* = 4,482). Moreover, trainees who completed their training in less than 13 months were excluded, as training completions within the first training year are atypical (e.g., recognition of prevocational programs). These adjustments led to the elimination of 177 cases. The dataset includes trainees from different training occupations. In detail, we examine 151 different professional groups (based on ISCO-08 categorization). In accordance with the structure of the dual system in Germany, these are primarily craft, production and service occupations. Although we impute some of the variables (see Section “Analysis Strategy”), cases with missing values for the educational qualification variable are excluded (*n* = 210). Missing values for this variable is often associated with missing values for other variables relevant to educational pathways in the NEPS dataset (grades and educational discontinuity) and limit the possibility of imputation. After the adjustment, 4,097 cases remained.

### Dependent Variables

#### Training Satisfaction

The first dependent variable is *training satisfaction*. Trainees reported their satisfaction with the training program on an 11-point Likert scale (0: ‘‘completely dissatisfied’’ to 10: ‘‘completely satisfied’’) during annual interviews after leaving general education. We use the last known information on training satisfaction, which is assigned to the survey wave of the first VET program by the data provider LifBi. For this variable, there were a large number of missing values (*n* = 1.995, 48.7% of the dataset). The share of missing values was especially high for trainees who terminated their training contract prematurely (*n* = 314, 58.6% of trainees with PCTs).^2^ We reduced the dataset for this analysis to trainees without missing values. Table 1 shows that the sample’s characteristics in the reduced dataset do not differ significantly from the original dataset except for educational certificates. This can be explained by a higher amount of missing values for trainees with PCT, who are more often low-skilled. The variable *training satisfaction* is right censored with a mean of 7.880 and a standard deviation of 1.690. This means that most trainees in the NEPS dataset reported rather high training satisfaction, which is in line with former studies (e.g., Volodina et al., 2015, p. 15).

**TABLE 1:** Distribution of independent and control variables.

	Total sample	Successful in first training year	Unsuccessful in first training year	With information on training satisfaction
General characteristics (in%)				
No or low school-leaving qualification	31.8	29.6	46.3	27.5
Intermediate school-leaving qualification	48.2	49.2	41.4	39.2
High school-leaving qualification	20.1	21.2	12.3	33.3
Entering preferred occupation	27.0	27.9	20.5	23.7
Graduation in typical number of years of general education	78.8	79.9	71.3	72.5
Participation in prevocational program before training	18.2	17.0	26.3	19.3
Sex (female)	40.4	39	49.3	43.8
Migration background	21.8	20.5	30.2	22.4
Low parental education	10.1	10.3	8.2	9.0
Company size < 10	22.7	20.5	37.0	22.3
Regional hiring challenges	5.8	5.8	5.9	5.6
GPA & self-efficacy (mean/standard deviation)				
GPA	2.675/0.535	2.656/0.530	2.810/0.546	2.719/0.512
Self-efficacy (z.-stand.)	0/1	0.007/0.992	−0.044/1.052	−0.003/1.029
Personal characteristics (z.-stand., mean/standard deviation)				
Realistic interests	0/1	0.036/1.001	−0.259/0.957	−0.075/0.987
Investigative interests	0/1	0.006/1.001	−0.043/0.996	−0.009/0.992
Artistic interests	0/1	−0.038/0.987	0.274/1.053	0.047/0.992
Social interests	0/1	−0.030/0.991	0.216/1.039	0.054/0.985
Enterprising interests	0/1	−0.015/0.999	0.106/1.004	0.030/0.981
Conventional interests	0/1	0.005/1.001	−0.040/0.992	0.039/0.974
Mathematical competence	0/1	0.042/1.007	−0.295/0.895	0.045/1.032
Scientific literacy	0/1	0.024/0.999	−0.169/0.994	0.031/1.012
Reading competence	0/1	0.008/1.001	−0.056/0.986	0.063/1.023
Vocational environment (z.-stand., mean/standard deviation)				
Share of low-qualified beginning trainees in occupation	0/1	−0.058/0.989	0.406/0.980	−0.143/0.988
Relevance of Realistic interests	0/1	0.018/1.010	−0.113/0.925	−0.093/1.025
Relevance of Investigative interests	0/1	0.037/0.997	−0.240/0.986	−0.020/1.027
Relevance of Artistic interests	0/1	−0.046/0.939	0.299/1.290	0.006/1.018
Relevance of Social interests	0/1	−0.045/0.985	0.292/1.049	0.072/1.037
Relevance of Enterprising interests	0/1	−0.029/0.996	0.187/1.006	0.049/0.999
Relevance of Conventional interests	0/1	0.000/1.008	−0.003/0.945	0.059/1.016
Importance of mathematical competence	0/1	0.008/1.007	−0.054/0.950	0.031/1.039
Importance of scientific literacy	0/1	0.013/0.990	−0.085/1.061	0.009/1.037
Importance of reading competence	0/1	0.008/0.992	−0.052/1.049	0.104/1.016

*Data not imputed.*

#### Premature Contract Termination

The variable premature contract termination (PCT) indicates whether trainees successfully completed the first 12 months of training (PCT = 0) or whether a premature termination occurred during the first 12 months of training (PCT = 1). In our sample, 536 individuals terminated their training contract prematurely within the first 12 months of training.

### Independent Variables

#### Educational Match

To examine the role of educational match, the fit between the level of school-leaving certificate and the dominant school-leaving certificate to obtain a training position in the respective occupation is modeled. We distinguish three educational levels: (1) no or low school-leaving qualification (*Hauptschulabschluss*), (2) intermediate school-leaving qualification (*mittlerer Bildungsabschluss*), and (3) high school-leaving qualification (university entrance qualification; *Fachhochschulreife/Abitur*). To investigate the effects of educational match, educational achievement before training is matched with information on the distribution of school-leaving qualifications from official data on beginners in dual VET programs for different occupations in Germany (Statistisches Bundesamt, n. d.). Previous research shows, that PCT pertains mainly to trainees with low qualification (Beicht and Ulrich, 2009, p. 30; Rohrbach-Schmidt and Uhly, 2015, p. 128 f.). Therefore, we concentrate on two distinct conditions: undereducation of trainees with low qualification and overeducation of trainees with intermediate and high school-leaving qualification. We generate a variable that measures the share of trainees with either no or low school-leaving qualification. We call this variable *share of low-qualified beginning trainees in occupation* (mean across all occupations = 31.5%; SD = 23.7%; min = 0%; max = 100%). This variable is used to analyze the influence of overeducation in interaction with either intermediately or highly qualified trainees (see Section ‘‘Analysis Strategy’’). Due to the intermediate qualification being the standard qualification level in VET, we refrain from distinguishing intermediately and highly qualified trainees with respect to overeducation. To investigate the effects of undereducation, we use the *share of at least intermediately qualified beginning trainees in occupation* (see Section ‘‘Analysis Strategy’’). Official data about the school-leaving qualifications of beginning trainees are published annually. Each NEPS participant is matched with official data depending on the starting year of dual training.^3^ Both variables of the qualification level in trainees’ occupations are used in a z-standardized form.

#### Interest Congruence

Interactions between trainees’ self-assessment of vocational interests before^4^ starting a training program and expert ratings of relevant vocational interests for trainees’ occupations are modeled based on the RIASEC model. In the NEPS dataset, trainees’ self-assessment for each dimension of the RIASEC model is based on items of two test instruments with three questions per dimension (*Realistic* and *Artistic*: revision of the Inventory of Children’s Activities [ICA-D], von Maurice, 2006, German version of ICA-R, Tracey and Ward, 1998; *Investigative*, *Social*, *Enterprising* and *Conventional*: AIST-R test instrument, Bergmann and Eder, 2005). We used trainees’ self-assessment of vocational interests of the last survey wave in general education before starting the VET program. All items were measured on a 5-point Likert scale. Reliability (Cronbach’s alpha) ranged from moderate to good: Realistic: 0.743; Investigative: 0.673; Artistic: 0.681; Social: 0.757; Enterprising: 0.525; and Conventional: 0.589. In particular, the scale of Enterprising has a low reliability, which could not be improved *via* item elimination and is probably due to the small number of items (Taber, 2018). For the analysis, the average per dimension is calculated, and all scales are z-standardized. Expert ratings are obtained from the O*NET interest database (O*Net, n.d.a).^5^ The RIASEC expert ratings are available on a 7-point Likert scale and also used in z-standardized form.

#### Skill Congruence

The NEPS dataset provides information about competence/literacy in mathematics, reading comprehension and science. All competence/literacy scales were Rasch scaled (WLE Reliability: 0.794 for mathematic, Duchhardt and Gerdes, 2013; 0.749 for reading comprehension, Haberkorn et al., 2012; 0.777 for science, Schöps and Sass, 2013). These skills were measured in 9th grade and are provided as Rasch scaled robust estimators for a person’s ability (Warm’s mean weighted likelihood estimates [WLE]). Corrected scales were used.^6^ Again, expert ratings are obtained from the O*NET database (O*Net, n.d.b). Here, experts rated the importance of all three skill dimensions on a 5-point Likert scale. For matching we again used the above described procedure.

### Control Variables

As control variables, we, first, include a variable indicating whether participants’ occupation matches the occupation they intended to apply to before starting a training program on the 5-digit level of the KldB 2010 (German classification system of occupations). As several studies have found significant effects of educational discontinuities and GPA on PCT (e.g., Beicht and Ulrich, 2009, p. 30; Beicht and Walden, 2013, p. 6), we control for the fact whether the final school-leaving certificate was achieved in the standard number of years of general education (low school-leaving qualification: 9 years, intermediate school-leaving qualification: 10 years, high school-leaving qualification: 12–13 years). As Busse (2020, p. 130) shows for school leavers, a later beginning of a VET program occurs almost directly after participating in prevocational programs. Therefore, a second variable measures whether adolescents have participated in prevocational programs prior to their training program. Additionally, students’ grade point average (GPA) of the last known school-leaving certificate is used as a control variable. Based on Lent and Brown (1996), we also control for self-efficacy. According to Bandura (1994), individuals with high self-efficacy believes are more capable to deal with setbacks and failures; they perceive difficult tasks as challenges instead of avoiding them. For Swiss trainees, Findeisen et al. (2022) show that occupational self-efficacy positively predicts persistence intention of trainees in VET. For self-efficacy, the mean of a 10-item scale is used in z-standardized form (Cronbach’s alpha: 0.799). The construct was measured in the years 2012/2013 (wave 5). Previous research shows also migration-related and social disparities in VET (Seeber et al., 2019b). Therefore, control variables measure trainees’ migration background (indicating whether the trainee or at least one parent was born abroad (=1) or not (=0)) as well as parents’ education (1: low educational background; both parents achieved at most a lower secondary education; 0: at least one parent achieved an intermediate or high school-leaving qualification). For the analysis of parental background, we used the last information before starting the VET program. Another control variable distinguishes whether the trainee is female (=1) or male (=0). Furthermore, we control for the size of the training company, which is dichotomized (1: small company [<10 employees] or 0: larger company [> = 10 employees]). Larger companies are expected to be more experienced in training adolescents and to have higher-quality training programs (e.g., quality of trainers, structured programs, etc.). Research found higher rates of PCT for small companies with less than 10 employees (e.g., Rohrbach-Schmidt and Uhly, 2016, p. 388). All information of the previous described control variables is based on trainees’ self-reports. Due to regional context differences (Michaelis and Busse, 2021), we control for local hiring challenges in the German training market. We use the proportion of vacant training positions to all offered training positions in the dual system (at the level of employment agency district).^7^ Additionally, the federal state in which participants’ training takes place is included as dummy variable in our models (15 variables, one reference variable). Publishing these effects is prohibited by the NEPS license agreement. All control variables are considered in each of the regression models. Table 1 shows the distribution of independent and control variables. In addition, a correlation matrix is provided in the Supplementary Material.

### Analysis Strategy

To determine the predictors of training satisfaction, Tobit regressions are used to handle the right-censored data. Binomial logistic regressions are used to analyze PCT in the first year of training. We report average marginal effects (AME), which indicate the average change in the probability of PCT in percentage points if an independent variable increases by one unit. We use interactions to measure the influence of educational match, interest and skill congruence. Interactions are characterized by main variables with information on the person and his/her environment (e.g., trainees’ Realistic interests and relevance of Realistic interests in trainees’ occupation) and an interaction term that contains the product of the main variables. The interaction term provides information on whether, for example, the vocational environment moderates the relationship between the trainees’ characteristics and the independent variables.

The influence of educational match on training satisfaction as well as PCT is examined in two separate, analogously structured analyses. Two models analyze the influence of under- and overeducation (M1 and M2). Undereducation is determined by an interaction term between the variable *no or low school-leaving qualification* and the variable *share of beginning trainees with at least intermediate qualification in trainees’ occupations*. Overeducation is investigated using two interaction terms: (1) intermediate school-leaving qualification and (2) high school-leaving qualification and share of low-qualified beginning trainees in trainees’ occupations. The models for testing an educational match also control for trainees’ vocational interests, skills, and the realization of the preferred occupation.

Second, the influence of vocational interest and skill congruence on training satisfaction as well as PCT is examined. Interaction terms are formed for each RIASEC dimension between trainees’ vocational interests and variables about the relevance of vocational interest dimensions for trainees’ occupations (M3). For the analysis of the effects of skill congruence, we proceed similarly. We integrate the interaction terms between trainees’ skills and the importance of each skill for trainees’ occupations (M4). All models also control for trainees’ educational background. It is important to note, that interaction terms have to interpreted with caution, because their significance only indicates a moderating effect between the main variables. In some cases, the direction of the effect could change due to the influence of main variables in the regression. For significant interaction terms, we estimate predicted probabilities and visualize the effect for a better understanding. According to recommendations for P-E fit measurement, we used polynomial regressions coefficients with interactions terms (Edwards and Parry, 1993). Multiple imputations using chained equations (n = 10) are performed, including all variables as predictors. The analyses are performed in Stata 16.

## Results

### Effects of Educational Match

**FIGURE 2:**
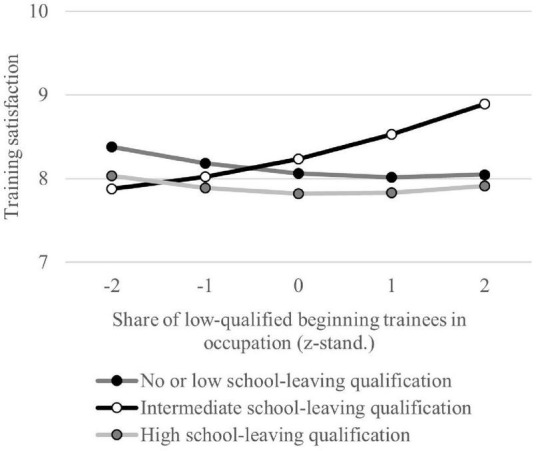
Training satisfaction based on school-leaving certificate and share of trainees with low qualification in the occupation. Based on regression model M2a. The share of low-qualified beginning trainees in trainees’ occupations is z-standardized.

Table 2 contains the regression results for analyzing the influence of educational match on training satisfaction. The interaction effect to investigate undereducation in M1a is not significant (supporting H1a). The results in M2a show that accounting for the interaction of trainees with an intermediate school-leaving qualification and the share of low-qualified beginning trainees in an occupation contravenes the general validity of H1a. Moderate overeducation has a positive effect on training satisfaction. Since the main effects (educational level and share of low-qualified beginning trainees in occupation) are not significant, only the change in training satisfaction should be interpreted based on the share of low-qualified beginning trainees in the occupation. Figure 2 visualizes interaction effects. It becomes clear that the training satisfaction of trainees with an intermediate school-leaving qualification increases by a higher share of low-qualified beginning trainees in the respective occupation in comparison to trainees with other school-leaving qualifications. Additionally, trainees with a high school-leaving qualification show significantly lower training satisfaction. This indicates an effect of overeducation because most trainees in the German VET system have an intermediate school-leaving qualification and is in line with H1a.

**TABLE 2:** Regression models to explain training satisfaction/PCT by educational match.

	Tobit regression models to explain training						Logistic regression models to explain					
	satisfaction by educational match						explain PCT by educational match					
				
	M1a (undereducation)			M2a (overeducation)			M1b (undereducation)			M2b (overeducation)		
	b	SE	*p*-value	b	SE	*p*-value	AME	SE	*p*-value	AME	SE	*p*-value
No or low school-leaving qualification	−0.151	0.136	0.265				**0.040**	0.017	0.015			
Intermediate school-leaving qualification				0.172	0.136	0.205				−**0.040**	0.015	0.010
High school-leaving qualification	−**0.254**	0.123	0.038	−0.242	0.182	0.184	−**0.043**	0.015	0.003	−**0.061**	0.018	0.001
Share of low-qualified beginning trainees in occupation				−0.083	0.115	0.468				**0.038**	0.014	0.007
Share of at least intermediate-qualified beginning trainees in occupation	−**0.161**	0.068	0.019				−**0.023**	0.009	0.011			
No or low school-leaving qualification × share of at least intermediate-qualified beginning trainees in occupation	0.259	0.134	0.054				−0.016	0.015	0.279			
Intermediate school-leaving qualification × share of low-qualified beginning trainees in occupation				**0.337**	0.140	0.016				−0.021	0.015	0.164
High school-leaving qualification × share of low-qualified beginning trainees in occupation				0.053	0.168	0.755				0.014	0.023	0.555
Constant term	**8.030**	0.380	0.000	**7.895**	0.389	0.000						
R^2^/Pseudo R^2^	0.051			0.052			0.084			0.085		
*n*	2,102						4,097					

*Effects with an p < = 0.05 in bold type; to allow easier interpretation of the effect of overeducation, the reference category for educational achievement in M2a/M2b has been changed; extended regression models in Supplementary Material (A2 and A3).*

Table 2 also presents the regression results for analyzing the influence of educational match on PCT. We find a significant effect of the school-leaving qualification. The predicted probability of PCT for a trainee with no or a low school-leaving certificate is 19.05% in comparison to 11.25% for an intermediate school-leaving qualification or 8.03% for a high school-leaving qualification (margins calculation based on M0b in Supplementary Material). The interaction terms for differentiated information of undereducation (M1b) and overeducation (M2b) are not significant. This means, for example, that—in line with H1b—the risk of PCT does not increase further for a trainee with a low school-leaving qualification if the trainee’s occupation is predominantly characterized by persons with at least an intermediate school-leaving qualification. Nevertheless, the high risk of PCT for trainees with no or a low school-leaving qualification indicates a general effect of undereducation that is not influenced by educational qualification in the occupation.

### Effects of Interest Congruence

In regard to the effects of interest congruence on training satisfaction (Table 3: M3a), there is only a generally significant positive influence of trainees’ Realistic interests. The variable indicating the relevance of Realistic interests for the particular occupation as well as the relating interaction term, however, are not significant. Hence, H3a is not supported.

**TABLE 3:** Regression models to explain training satisfaction/PCT by interest and skill congruence.

	Tobit regression models to explain training						Logistic regression models to explain PCT					
	satisfaction by interest and skill congruence						by interest and skill congruence					
				
	M3a (+ interaction			M4a (+ interaction			M3b (+ interaction			M4b (+ interaction		
	terms RIASEC)			terms competences)			terms RIASEC)			terms competences)		
	b	SE	*p*-value	b	SE	*p*-value	AME	SE	*p*-value	AME	SE	*p*-value
Realistic orientation	**0.132**	0.061	0.031	**0.139**	0.059	0.018	−**0.021**	0.008	0.008	−**0.032**	0.008	0.000
Investigative orientation	−0.030	0.059	0.609	−0.038	0.058	0.514	0.000	0.007	0.946	−0.001	0.007	0.907
Artistic orientation	−0.033	0.058	0.570	−0.036	0.052	0.489	**0.026**	0.008	0.001	**0.030**	0.006	0.000
Social orientation	0.045	0.058	0.439	0.029	0.056	0.606	0.006	0.007	0.402	0.009	0.007	0.195
Enterprising orientation	−0.032	0.058	0.585	−0.027	0.057	0.634	**0.016**	0.007	0.024	**0.015**	0.007	0.035
Conventional orientation	0.039	0.059	0.508	0.039	0.059	0.512	−0.008	0.007	0.208	−0.008	0.006	0.208
Relevance of Realistic interests	−0.033	0.183	0.858				0.025	0.022	0.244			
Relevance of Investigative interests	0.003	0.090	0.978				−**0.037**	0.010	0.000			
Relevance of Artistic interests	0.030	0.085	0.721				**0.028**	0.010	0.005			
Relevance of Social interests	0.003	0.116	0.981				**0.032**	0.013	0.016			
Relevance of Enterprising interests	−0.103	0.152	0.504				0.005	0.017	0.780			
Relevance of Conventional interests	−0.076	0.097	0.437				0.005	0.011	0.650			
Realistic orientation × relevance of Realistic interests	−0.030	0.065	0.642				−**0.017**	0.007	0.024			
Investigative orientation × relevance of Investigative interests	−0.013	0.055	0.810				0.002	0.006	0.711			
Artistic orientation × relevance of Artistic interests	−0.052	0.048	0.281				−0.010	0.005	0.053			
Social orientation × relevance of Social interests	−0.006	0.055	0.915				−0.001	0.006	0.918			
Enterprising orientation × relevance of Enterprising interests	0.014	0.049	0.779				−0.001	0.006	0.836			
Conventional orientation × relevance of Conventional interests	−0.054	0.051	0.293				−0.003	0.006	0.613			
Mathematical competence	0.023	0.062	0.710	0.017	0.067	0.797	−0.017	0.007	0.021	−**0.016**	0.008	0.036
Scientific literacy	−**0.176**	0.065	0.007	−**0.179**	0.068	0.009	0.011	0.008	0.150	0.008	0.008	0.263
Reading competence	−0.012	0.061	0.843	−0.023	0.063	0.716	**0.019**	0.008	0.017	**0.021**	0.008	0.011
Importance of mathematical competence				−0.045	0.055	0.414				0.007	0.006	0.287
Importance of scientific literacy				0.046	0.062	0.457				−**0.018**	0.007	0.014
Importance of reading competence				−0.113	0.063	0.072				−**0.022**	0.007	0.001
Mathematical competence × importance of mathematical competence				−0.005	0.045	0.909				0.000	0.007	0.992
Scientific literacy × importance of scientific literacy				0.001	0.042	0.982				−0.006	0.005	0.284
Reading competence × importance of reading competence				−0.061	0.051	0.235				−0.006	0.005	0.307
Constant term	**7.837**	0.405	0.000	**7.882**	0.383	0.000						
R^2^/pseudo R^2^	0.060			0.056			0.103			0.086		
*n*	2,102						4,097					

*Effects with an p < = 0.05 in bold type; extended regression models in Supplementary Material (A4 and A5).*

**FIGURE 3:**
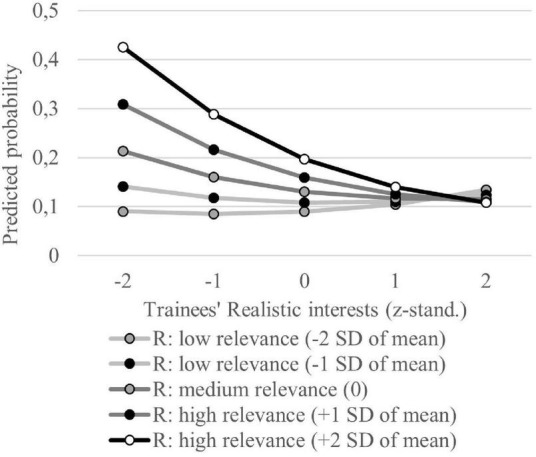
Probability of PCT depending on trainees’ Realistic interests for different Realistic (R) relevance levels in trainees’ occupations. Based on regression model M3b. Realistic interests are z-standardized. Values of Realistic relevance levels in trainees’ occupations are based on z-standardized expert ratings.

**FIGURE 4:**
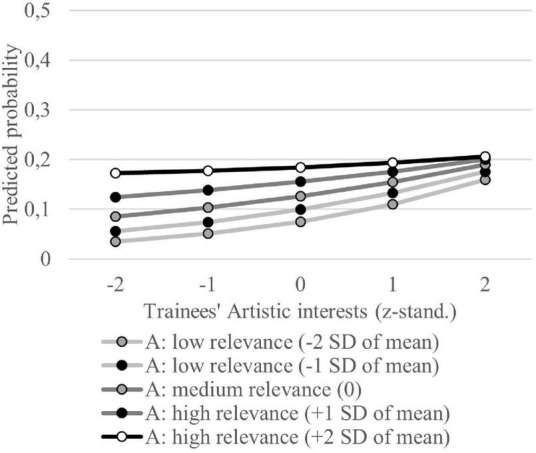
Probability of PCT depending on trainees’ Artistic interests for different Artistic (A) relevance levels in trainees’ occupations. Based on regression model M3b. Artistic interests are z-standardized. Values of Artistic relevance levels in trainees’ occupations are based on z-standardized expert ratings.

Regarding PCT (Table 3: M3b), several RIASEC-related variables show significant effects. Higher values in the Artistic and Enterprising dimensions increase the probability of PCT. Only occupations that require Investigative interests have a lower probability of PCT. When interaction terms between the trainees’ interests and the required interests for occupations are modeled (M3b), we find significant interaction terms for the Realistic and the Artistic dimension. Both interaction terms are negative. Nevertheless, the results of both constructs differ clearly because of differences in the main effects (see Figures 3, 4). For the Realistic dimension, the importance of the congruence between trainees’ interest and interests attributed to the occupation becomes visible. The visualization of the Artistic interest congruence shows that no congruence effect exists. The probability of PCT is always high in Artistic interest-related occupations independent of trainees’ Artistic interests. Here, the interaction term becomes significant because the probability functions differ by the relevance of Artistic interest in the respective occupation. This means that for occupations where Artistic interests are less relevant, an increase in trainees’ Artistic interests leads to a higher increase in PCT probability than in occupations where this disposition is of higher relevance. Nevertheless, it becomes clear that high Artistic interests are problematic in all occupations and can foster the probability of PCT. The effect of Enterprising interests on PCT shows a similar result but without an influence of the relevance of the vocational environment. Hence, H3b holds only for Realistic interests.

### Skill Congruence

For the explanation of training satisfaction as well as PCT, the results in Table 3 (M4b) do not suggest any influence of skill congruence (contradicting H4a and H4b). Only single main effects are significant. Higher values of scientific literacy reduce training satisfaction. In contrast, higher values of mathematical competences have a preventive effect on PCT, and higher reading competences can increase the probability of PCT.

## Discussion

### General Discussion

The aim of this contribution is to analyze the influence of selected indirectly and objectively measured P-V fit indicators (educational match, vocational interest congruence, and vocational skill congruence) on training satisfaction and PCT. As the results show, only individual P-V fit indicators and individual variables of the P-V fit interaction terms are significant. This means that for most interest and all skill indicators examined, trainees with congruent and incongruent P-V fit do not differ with regard to both training satisfaction and PCT. Thus, for the context of VET, objective incongruences regarding P-V fit does not lead to negative consequences for the training process. This contradicts the existing results from career research. An explanation for the deviation in those findings could lie in the particularities of VET programs. In VET, work-adjustment processes are highly structured and closely accompanied by supervisors. Additionally, the training process in Germany is accompanied by learning phases in vocational schools. These aspects could facilitate settling in at the workplace. However, in the VET context, there are hardly any findings regarding work-adjustment processes, in particular the influence of organizational socializing tactics and proactive behavior by trainees during the introductory phase of training.

Our results also suggest that trainees’ *perception* of fit might be more important than objectively determined fit, as prior studies find significant positive effects of *perceived* PV-fit on persistence intention (Nägele and Neuenschwander, 2015; Findeisen et al., 2022).

Other specific results are also not in line with our hypotheses. In regard to educational mismatch, we find that overeducation does not always lead to negative effects on training satisfaction or PCT, as suggested by the existing research literature (Sánchez-Sánchez and McGuinness, 2015; Verhaest and Verhofstadt, 2016). As our results show, moderate overeducation in the case of an intermediate school-leaving qualification can even increase training satisfaction for occupations with a high share of trainees with low qualifications. We assume that trainees with an intermediate school-leaving qualification more easily cope with vocational demands than low-skilled trainees. This can positively influence the experience of competence and, hence, increase satisfaction. The result that trainees with high school-leaving qualification show significantly reduced training satisfaction independent of qualification level in their occupation is more in line with general findings on overeducation (Sánchez-Sánchez and McGuinness, 2015; Verhaest and Verhofstadt, 2016). Explanations could lie, for example, in limited possibilities to realize one’s abilities and skills. The general risk of low qualification for PCT is in line with previous research in the context of VET (Beicht and Ulrich, 2009, p. 30; Rohrbach-Schmidt and Uhly, 2015, p. 128 f.).

Regarding interest congruence, our results generalize findings of Volodina et al. (2015, p. 18) that Realistic interest congruence is important for PCT in the early career stage. Apart from occupations characterized by Realistic interests our results reveal that interest incongruences neither influence training satisfaction nor PCT. Several studies verified that interests are trait factors which mean they are rather stable over time (Tracey and Sodano, 2008; Wille and De Fruyt, 2019). However, Wille et al. (2014, p. 64) show that Realistic interests have the strongest re-test reliability in the first 15 years of a career (Realistic: 0.76; Investigative: 0.51; Artistic: 0.50; Social: 0.49; Enterprising: 0.37; Conventional: 0.43). Thus, our results could indicate interest adjustments in the first year of VET so that most incongruences do not strain the training process.

With respect to skill congruence, none of the competence measures examined (mathematical competence, reading competence and science literacy) had a significant effect on training satisfaction or PCT. This result, again, generalizes the findings of Volodina et al. (2015, p. 18). However, this does not mean that skills are not relevant for the training process as the general effect of mathematical skills on PCT indicate. Instead, the finding shows that mathematical skills are important for all vocations. This is also an important implication for research in competence development in VET, where theory suggested that basal skills play a central role in coping with training requirements in general (Winther and Achtenhagen, 2009, p. 92).

In addition to the general effect of mathematical skills, the results show that further certain individual characteristics and dispositions of trainees are relevant to predicting training satisfaction and PCT, regardless of the characteristics of the vocational environment. Some dispositions of trainees lead to negative consequences in the training process: Enterprising interests as well as reading competence affect PCT, and scientific literacy leads to lower training satisfaction. It can be assumed that high levels in these dimensions cannot be applied in occupations within the vocational structure of the German dual system. Adolescents with these characteristics may aspire more to occupations that can be pursued only through higher education. This could also be true for strong Artistic interests, which lead to high probabilities of PCT in all occupations.

Our findings are also important for research analyzing transition from school to VET and relating implications to the relevance of using adolescents’ signals to secure P-V fit by training providers in VET. Relating studies show that adolescents with better qualification (e.g., Holtmann et al., 2017; Michaelis and Busse, 2021) and school achievement in mathematics and mother tongue (e.g., Eberhard, 2012; Beicht and Walden, 2018) find it easier to achieve a training position in competing training markets like German dual system. This indicates that companies should use such information as signals for adolescents’ potential training performance. Our findings support the relevance of signaling for selecting trainees against the background of the general effects of low-qualification and mathematical skills. However, our findings also reveal that several potential signals are not that important and some signals like high educational qualification or strong reading skills could also lead to frictions in the training process.

### Limitations

The analysis contains certain limitations that should be taken into account. Due to the higher proportion of missing values on the variable training satisfaction among trainees with PCT, it is unfortunately not possible to model complex structural relationships between P-V fit indicators, training satisfaction and PCT, as is assumed by work-adjustment theory (Dawis and Lofquist, 1984). Additionally, training satisfaction has been measured only by one general variable. Thus, it is not possible to estimate reliability coefficients. Additionally, we could not distinguish between satisfaction with the training company and the vocational school, because this information is only available for successful trainees. Different effects could occur, if trainees are not equally satisfied with both training institutions. Furthermore, measurement of training satisfaction in NEPS is a wave-specific measurement and particularly frequently affected by missing values. Etzel and Nagy (2021) show for industrial clerks and four occupations of technical trainees that training satisfaction decreased in all occupations during the training process. Only in occupations with stronger increase in interest congruence they find a smaller reduction of training satisfaction. However, based on our data, we cannot examine the influence of P-V fit on the *development* of training satisfaction during a specific training program.

We used an indirect measurement assessing personal and environmental characteristics separately. Hereby, information on environmental characteristics are obtained objectively based on the O*NET database. In general, there are different assessment methods to measure P-E fit (and thus also P-V fit) (Kristof-Brown et al., 2005). As no direct measurements with a subjective evaluation of the P-E fit (perceived P-E fit) or subjective assessments of the vocational environment exist in the NEPS dataset, no comparisons can be made to different forms of P-E fit measurement. Hence, we cannot examine, to what extent our results are influenced by the measurement approach for P-V fit.

Moreover, skill variables were measured in 2010. While most trainees started their training in 2012, some entered a training program at a later point in time (2016 at the latest). As skills develop, the skill values in the NEPS dataset can be seen only as an indicator of skill potential. This might explain the non-significant effects regarding skill congruence.

Furthermore, explained variance in our models is rather low due to the limited set of control variables used. However, Böhn and Deutscher (2021) show by a systematic literature review that PCT is affected by 68 predictors. The NEPS dataset is in particular limited to training company information. Consequently, there is quite a large number of additional predictors that affect PCT and could not have been considered in this study. Additionally, our analysis did not consider multilevel structures. While it has been shown for PCT that multilevel analyses could enhance analysis quality (Rohrbach-Schmidt and Uhly, 2015), the requirements for such methods are strong. Hox (1998) recommends at least 50 groups and 20 individuals per group for cross-level interactions (such as the interactions in this study). We have 151 ISCO-08 groups (4-digit level), but 107 occupations (70.9%) have fewer than 20 cases, and some have only a few individual cases. Moreover, missing values in the variable training satisfaction increases this problem. Thus, the NEPS dataset does not meet the requirements of a multilevel analysis regarding P-V fit. Finally, although the NEPS dataset is a longitudinal dataset, the survey design with only annual survey waves unfortunately does not allow for a more differentiated analysis of work-adjustment processes. This would require more frequent re-interviews during the VET program.

### Implications for Future Research and Practice

Against the background of our results, two related research fields should be analyzed further. Etzel and Nagy (2021) already reveal that vocational interest congruence is relatively stable over the training process and thus socialization effects are low. However, more differentiated effects of work-adjustment processes and educational mediation in the field of VET are of interest. For instance, it should be examined how different types of training quality affect the development of skill congruence, training satisfaction as well as PCT. On the other hand, differentiated analyses of the subsequent employment of trainees with PCT in interaction with P-V fit and its development should be pursued. Research on PCT—as well as research on dropout or turnover in general—typically falls short in regard to the inclusion of further career paths of adolescents who terminate their training contract prematurely. Depending on the subsequent employment or status of each individual, issues of P-V fit can be evaluated more thoroughly. Furthermore, while our study focused on explaining satisfaction and PCT, it might be worthwhile to analyze the influence of P-V fit on other indicators of training success, such as professional competence during the training program, success in final exams or career path after completing the training program.

More generally, there seem to be different mechanisms at work in regard to explaining satisfaction as well as PCT in the field of VET compared to other work contexts (e.g., Bowling and Hammond, 2008) or higher education programs (e.g., Lent et al., 2016). It seems worthwhile for future research endeavors to examine differences more deeply to see which effects are transferable across contexts and which effects are context-specific.

For practical implications, it is important to mention that most objectively measured P-V incongruences do not lead to negative consequences for the training process. This indicates that for the transition from school to VET a lack of fit is not problematic in general. For providers of training programs our results reveal that a certain risk can be taken in hiring decisions for trainees. Nevertheless, where Realistic interests are relevant, companies should pay attention to this disposition in the recruitment process. Furthermore, training strategies for trainees with high school-leaving qualification should be critically reflected to increase the satisfaction of these trainees.

## Data Availability Statement

Publicly available datasets were analyzed in this study. This data can be found here: doi: 10.5157/NEPS:SC4:10.0.0, Leibniz Institute for Educational Trajectories (LIfBi).

## Ethics Statement

Ethical review and approval, and written informed consent to participate, were not required for the current study in accordance with the local legislation and institutional requirements. The analyses of this manuscript are secondary analyses of data published previously (Blossfeld et al., 2011). Data sources used for the analyses were the cohort ninth-grade students (doi: 10.5157/NEPS:SC4:10.0.0) of the German National Educational Panel Study. The NEPS study is conducted under the supervision of the German Federal Commissioner for Data Protection and Freedom of Information (BfDI) and in coordination with the German Standing Conference of the Ministers of Education and Cultural Affairs (KMK) and—in the case of surveys at schools—the Educational Ministries of the respective federal states. All data collection procedures, instruments, and documents were checked by the data protection unit of the Leibniz Institute for Educational Trajectories (LIfBi). Participation was only possible if adolescents gave informed consent to participate and their parents/guardians have consented in writing. All data analyses were performed via a remote desktop (RemoteNEPS) at the LIfBi (in Bamberg, Germany) that provided a controlled privacy environment for data access.

## Author Contributions

CM and SF contributed to the conception and the design of the study and edited the manuscript in several rounds. CM prepared the data, performed the statistical analysis, and visualized the figures and tables. Both authors read and approved the submitted and the final version.

## Conflict of Interest

The authors declare that the research was conducted in the absence of any commercial or financial relationships that could be construed as a potential conflict of interest.

## Publisher’s Note

All claims expressed in this article are solely those of the authors and do not necessarily represent those of their affiliated organizations, or those of the publisher, the editors and the reviewers. Any product that may be evaluated in this article, or claim that may be made by its manufacturer, is not guaranteed or endorsed by the publisher.

## Footnotes

1 An analysis based on the NEPS dataset that is also used for the purpose of our study (see Section “Materials and Methods”), shows that 48% of PCT are initiated by the trainee, 23% by the training company, and 29% based on mutual agreement of both parties.

2 This also limited the analysis potential. While an interaction between the types of fit and training satisfaction on PCT is theoretically plausible (cf. Dawis and Lofquist, 1984), interaction effects could not be analyzed here.

3 The KldB 2010 is used to merge official data on school-leaving qualification with the NEPS dataset. The KldB 2010 is the standard system for the classification of occupations in Germany (Paulus and Matthes, 2013). Official data about the school-leaving qualifications of beginning trainees are available only since 2012. Some trainees had already started their training in 2010 or 2011 (*n* = 476). In these cases, the NEPS data are matched with the official data for the training year 2012. Since the distributions within occupations are relatively stable over time, no significant bias should be expected.

4 Most traits in NEPS are not regularly surveyed. Participants indicated their vocational interests for the first time in the second survey wave (summer 2011). If adolescents did not leave general education before survey wave 8, they were re-interviewed regarding their vocational skills in survey wave 8 (2014/2015) and survey wave 10 (2016/2017). As most adolescents in our sample left general education before survey wave 8, we used the information of the second survey wave.

5 In O*NET, expert ratings are available according to Standard Occupational Classification (SOC) codes, in NEPS, occupations are classified by the International Standard Classification of Occupations of 2008 (ISCO-08, four-digit level). A conversion key between SOC and ISCO-08 codes (Bureau of Labor Statistics, 2019) was used. For cases in which the SOC code is more differentiated than the ISCO-08 code, the average for each RIASEC dimension is calculated over all values of an ISCO-08 occupation.

6 In NEPS, different competence domains have been assessed simultaneously. Therefore, “individual test scores are corrected for the position effect in the estimation of ability scores” (Pohl and Carstensen, 2012, p. 7).

7 For data protection reasons, these values are available only for 2012 in order to prevent the localization of NEPS participants. The potential bias should be low because 2012 is the median of the starting year for dual training of NEPS participants.
