# Supplementary material for: Influence of Person-Vocation Fit on Satisfaction and Persistence in Vocational Training Programs
Source: Front Psychol. 2022 Feb 14;13:834543. doi: 10.3389/fpsyg.2022.834543 (PMC8882963; doi:10.3389/fpsyg.2022.834543)
Supplement: Supplementary file 1 [file Data_Sheet_1.docx]

# Supplementary Material

*A1a Correlation matrix*

|  | (1) | (2) | (3) | (4) | (5) | (6) | (7) | (8) | (9) | (10) | (11) | (12) | (13) | (14) | (15) | (16) | (17) | (18) |
| --- | --- | --- | --- | --- | --- | --- | --- | --- | --- | --- | --- | --- | --- | --- | --- | --- | --- | --- |
| (1) Training satisfaction | 1.000 |  |  |  |  |  |  |  |  |  |  |  |  |  |  |  |  |  |
| (2) PCT | .011 | 1.000 |  |  |  |  |  |  |  |  |  |  |  |  |  |  |  |  |
| (3) No or low school-leaving qualification | **.121** | -.004 | 1.000 |  |  |  |  |  |  |  |  |  |  |  |  |  |  |  |
| (4) Intermediate school-leaving qualification | **-.052** | **.066** | **-.658** | 1.000 |  |  |  |  |  |  |  |  |  |  |  |  |  |  |
| (5) High school-leaving qualification | **-.075** | **-.065** | **-.342** | **-.483** | 1.000 |  |  |  |  |  |  |  |  |  |  |  |  |  |
| (6) Entering preferred occupation | **-.056** | **.048** | **.074** | .007 | **-.094** | 1.000 |  |  |  |  |  |  |  |  |  |  |  |  |
| (7) Graduation in typical number of years of general education | **-.071** | **.014** | **-.110** | **.159** | **-.071** | **.052** | 1.000 |  |  |  |  |  |  |  |  |  |  |  |
| (8) Participation in prevocational program before training | **.081** | **-.030** | **.363** | **-.180** | **-.197** | **.088** | **-.128** | 1.000 |  |  |  |  |  |  |  |  |  |  |
| (9) Sex (female) | **.070** | **.040** | **-.112** | **.010** | **.117** | **-.024** | **-.055** | **.011** | 1.000 |  |  |  |  |  |  |  |  |  |
| (10) Migration background | **.078** | **.034** | **.130** | **-.052** | **-.086** | **-.026** | **-.050** | **.104** | **.058** | 1.000 |  |  |  |  |  |  |  |  |
| (11) Low parental education | **-.024** | .007 | **.035** | **.029** | **-.076** | **.047** | **.045** | **-.021** | **.023** | .006 | 1.000 |  |  |  |  |  |  |  |
| (12) Company size < 10 | **.132** | **.025** | **.157** | **-.055** | **-.114** | **.018** | **-.042** | **.105** | **.132** | **.088** | .008 | 1.000 |  |  |  |  |  |  |
| (13) Regional hiring challenges | .009 | **.020** | **.028** | **.056** | **-.102** | **.011** | **.061** | **-.042** | .006 | **-.041** | **.017** | -.001 | 1.000 |  |  |  |  |  |
| (14) GPA | **.099** | **-.048** | **.157** | **-.172** | **.033** | **-.034** | **-.074** | **.147** | **-.085** | **.088** | **-.012** | **.054** | **-.069** | 1.000 |  |  |  |  |
| (15) Self-efficacy | -.017 | **.132** | **.071** | -.009 | **-.100** | .029 | -.021 | -.027 | **-.128** | **-.036** | -.020 | .004 | **-.042** | **-.050** | 1.000 |  |  |  |
| (16) Realistic interests | **-.096** | **.057** | **.037** | .016 | **-.058** | **.055** | **.081** | **-.033** | **-.457** | **-.081** | -.024 | **-.070** | **-.042** | .017 | **.155** | 1.000 |  |  |
| (17) Investigative interests | -.016 | -.003 | **-.072** | .024 | **.047** | .003 | .024 | -.009 | **-.134** | .023 | -.018 | -.025 | -.010 | **-.062** | **.046** | **.374** | 1.000 |  |
| (18) Artistic interests | **.102** | .006 | **-.058** | .015 | **.042** | **-.035** | -.020 | .048 | **.292** | **.096** | -.007 | **.079** | .012 | -.028 | .004 | **.068** | **.306** | 1.000 |
| (19) Social interests | **.080** | .030 | **-.043** | -.029 | **.079** | -.011 | **-.044** | .024 | **.388** | **.075** | .003 | **.093** | .005 | **-.040** | .007 | **-.138** | **.179** | **.342** |
| (20) Enterprising interests | **.039** | .013 | **-.073** | -.019 | **.099** | **-.054** | **-.037** | **-.032** | -.013 | **.057** | -.025 | -.008 | .013 | **-.040** | **.116** | **.057** | **.197** | **.160** |
| (21) Conventional interests | -.015 | .028 | **-.056** | -.023 | **.085** | -.020 | -.022 | -.024 | .004 | **.048** | -.015 | -.022 | .005 | **-.069** | **.054** | **.184** | **.322** | **.217** |
| (22) Mathematical competence | **-.111** | **-.049** | **-.329** | **.051** | **.299** | **-.045** | **.073** | **-.201** | **-.197** | **-.187** | -.024 | **-.164** | **.066** | **-.185** | **-.048** | **.175** | **.132** | **-.074** |
| (23) Scientific literacy | **-.064** | **-.089** | **-.336** | **.077** | **.274** | **-.043** | **.064** | **-.182** | **-.151** | **-.243** | **-.048** | **-.128** | **.035** | **-.163** | -.033 | **.145** | **.146** | -.026 |
| (24) Reading competence | -.021 | **-.055** | **-.380** | **.068** | **.331** | **-.031** | .018 | **-.182** | **.106** | **-.179** | **-.040** | **-.090** | **.040** | **-.197** | **-.091** | **-.053** | **.050** | **.047** |
| (25) Share of low-qualified beginning trainees in occupation | **.154** | **.051** | **.499** | **-.151** | **-.396** | **.063** | **-.041** | **.230** | **-.166** | **.085** | .030 | **.193** | .011 | **.227** | **.088** | **.151** | **-.036** | -.022 |
| (26) Relevance of Realistic interests | -.045 | .043 | **.180** | .013 | **-.236** | **.061** | **.052** | .017 | **-.508** | **-.038** | -.008 | -.017 | .000 | **.086** | **.085** | **.465** | **.139** | **-.130** |
| (27) Relevance of Investigative interests | **-.094** | .040 | **-.085** | **.108** | **-.037** | .005 | **.072** | **-.083** | **-.241** | **-.070** | -.018 | **-.050** | -.006 | **-.054** | .033 | **.275** | **.172** | **-.085** |
| (28) Relevance of Artistic interests | **.118** | .025 | **.070** | **-.054** | -.016 | **.090** | -.029 | **.053** | **.061** | .023 | -.014 | **.153** | .026 | .018 | .003 | .024 | -.021 | **.123** |
| (29) Relevance of Social interests | **.115** | .010 | **-.098** | .026 | **.086** | -.019 | **-.034** | .018 | **.577** | **.080** | .002 | **.162** | -.001 | **-.042** | **-.077** | **-.459** | **-.086** | **.156** |
| (30) Relevance of Enterprising interests | **.074** | -.045 | **-.042** | **-.057** | **.124** | **-.056** | **-.073** | **.058** | **.356** | **.065** | -.004 | **.043** | -.013 | .008 | -.032 | **-.351** | **-.124** | **.092** |
| (31) Relevance of Conventional interests | -.001 | **-.060** | **-.149** | -.020 | **.207** | **-.067** | **-.047** | -.008 | **.342** | .005 | .018 | **-.050** | -.027 | **-.090** | **-.068** | **-.365** | **-.100** | **.078** |
| (32) Importance of mathematical competence | -.021 | -.030 | **-.086** | -.015 | **.125** | .000 | -.020 | -.001 | -.025 | **-.038** | -.026 | **-.078** | -.022 | **-.037** | .018 | .005 | **.061** | -.013 |
| (33) Importance of scientific literacy | **-.033** | **.051** | **-.089** | **.124** | **-.052** | .017 | **.054** | **-.060** | -.020 | -.016 | -.017 | .019 | -.005 | **-.053** | .011 | **.107** | **.174** | -.008 |
| (34) Importance of reading competence | -.020 | -.030 | **-.275** | **.089** | **.220** | **-.083** | -.013 | **-.053** | **.426** | .007 | -.006 | **-.038** | -.010 | **-.125** | **-.069** | **-.337** | -.025 | **.099** |

*Note. effects with an p<=.05 in bold type*

*A1b Correlation matrix*

|  | (18) | (19) | (20) | (21) | (22) | (23) | (24) | (25) | (26) | (27) | (28) | (29) | (30) | (31) | (32) | (33) | (34) |
| --- | --- | --- | --- | --- | --- | --- | --- | --- | --- | --- | --- | --- | --- | --- | --- | --- | --- |
| (18) Artistic interests | 1.000 |  |  |  |  |  |  |  |  |  |  |  |  |  |  |  |  |
| (19) Social interests | **.342** | 1.000 |  |  |  |  |  |  |  |  |  |  |  |  |  |  |  |
| (20) Enterprising interests | **.160** | **.356** | 1.000 |  |  |  |  |  |  |  |  |  |  |  |  |  |  |
| (21) Conventional interests | **.217** | **.258** | **.455** | 1.000 |  |  |  |  |  |  |  |  |  |  |  |  |  |
| (22) Mathematical competence | **-.074** | **-.162** | **.044** | **.051** | 1.000 |  |  |  |  |  |  |  |  |  |  |  |  |
| (23) Scientific literacy | -.026 | **-.160** | .014 | **-.035** | **.606** | 1.000 |  |  |  |  |  |  |  |  |  |  |  |
| (24) Reading competence | **.047** | -.009 | .023 | -.029 | **.486** | **.570** | 1.000 |  |  |  |  |  |  |  |  |  |  |
| (25) Share of low-qualified beginning trainees in occupation | -.022 | **-.050** | **-.066** | **-.087** | **-.296** | **-.269** | **-.329** | 1.000 |  |  |  |  |  |  |  |  |  |
| (26) Relevance of Realistic interests | **-.130** | **-.236** | **-.108** | **-.078** | **.059** | **.069** | **-.148** | **.354** | 1.000 |  |  |  |  |  |  |  |  |
| (27) Relevance of Investigative interests | **-.085** | **-.119** | **-.090** | **-.047** | **.168** | **.165** | **.040** | **-.140** | **.575** | 1.000 |  |  |  |  |  |  |  |
| (28) Relevance of Artistic interests | **.123** | -.020 | **-.055** | **-.050** | -.010 | -.009 | .017 | **.203** | **.126** | **.054** | 1.000 |  |  |  |  |  |  |
| (29) Relevance of Social interests | **.156** | **.366** | **.050** | .001 | **-.160** | **-.152** | **.075** | **-.184** | **-.573** | **-.234** | -.015 | 1.000 |  |  |  |  |  |
| (30) Relevance of Enterprising interests | **.092** | **.141** | **.091** | **.067** | **-.098** | **-.106** | **.045** | **-.083** | **-.762** | **-.679** | **-.090** | **.404** | 1.000 |  |  |  |  |
| (31) Relevance of Conventional interests | **.078** | **.142** | **.086** | **.095** | .000 | -.012 | **.134** | **-.351** | **-.782** | **-.366** | **-.277** | **.307** | **.480** | 1.000 |  |  |  |
| (32) Importance of mathematical competence | -.013 | -.020 | .013 | .019 | **.101** | **.088** | **.076** | **-.129** | **-.109** | **.118** | -.006 | .020 | **.210** | **.116** | 1.000 |  |  |
| (33) Importance of scientific literacy | -.008 | **.041** | **-.076** | **-.043** | **.071** | **.078** | **.039** | **-.177** | **.458** | **.761** | -.018 | **.139** | **-.574** | **-.386** | **.141** | 1.000 |  |
| (34) Importance of reading competence | **.099** | **.205** | **.056** | **.055** | .012 | .003 | **.176** | **-.539** | **-.711** | **-.114** | **-.137** | **.557** | **.473** | **.542** | **.244** | **.054** | 1.000 |

*Note. effects with an p<=.05 in bold type*

*A2 Tobit regression models to explain training satisfaction by educational match (Extended model)*

|  | M0a | | | M1a (undereducation) | | | | M2a (overeducation) | | | |  |
| --- | --- | --- | --- | --- | --- | --- | --- | --- | --- | --- | --- | --- |
|  | b | S.E. | p-value | | b | S.E. | p-value | | b | S.E. | p-value | |
| No or low school-leaving qualification | -.183 | .122 | .133 | | -.151 | .136 | .265 | |  |  |  | |
| Intermediate school-leaving qualification |  |  |  | |  |  |  | | .172 | .136 | .205 | |
| High school-leaving qualification | **-.333** | .117 | .005 | | **-.254** | .123 | .038 | | -.242 | .182 | .184 | |
| Share of low-qualified beginning trainees in occupation |  |  |  | |  |  |  | | -.083 | .115 | .468 | |
| Share of at least intermediate-qualified beginning trainees in occupation |  |  |  | | **-.161** | .068 | .019 | |  |  |  | |
| No or low school-leaving qualification * share of at least intermediate-qualified beginning trainees in occupation |  |  |  | | .259 | .134 | .054 | |  |  |  | |
| Intermediate school-leaving qualification * share of low-qualified beginning trainees in occupation |  |  |  | |  |  |  | | **.337** | .140 | .016 | |
| High school-leaving qualification * Share of low-qualified beginning trainees in occupation |  |  |  | |  |  |  | | .053 | .168 | .755 | |
| Polynomial Coefficients |  |  |  | |  |  |  | |  |  |  | |
| No or low school-leaving qualification² |  |  |  | | .000 (omitted) |  |  | |  |  |  | |
| Intermediate school-leaving qualification² |  |  |  | |  |  |  | | .000 (omitted) |  |  | |
| High school-leaving qualification² |  |  |  | |  |  |  | | .000 (omitted) |  |  | |
| Share of low-qualified beginning trainees in occupation² |  |  |  | |  |  |  | | .037 | .052 | .468 | |
| Share of at least intermediate-qualified beginning trainees in occupation² |  |  |  | | .050 | .051 | .328 | |  |  |  | |
| Control Variables |  |  |  | |  |  |  | |  |  |  | |
| Entering preferred occupation | .155 | .105 | .138 | | .158 | .104 | .013 | | .147 | .104 | .160 | |
| Realistic orientation | **.163** | .056 | .004 | | **.153** | .057 | .008 | | **.154** | .057 | .007 | |
| Investigative orientation | -.025 | .056 | .660 | | -.028 | .056 | .062 | | -.022 | .056 | .692 | |
| Artistic orientation | -.036 | .051 | .478 | | -.037 | .051 | .468 | | -.038 | .051 | .456 | |
| Social orientation | .040 | .056 | .477 | | .040 | .056 | .475 | | .040 | .056 | .471 | |
| Enterprising orientation | -.033 | .057 | .560 | | -.034 | .057 | .558 | | -.034 | .057 | .548 | |
| Conventional orientation | .021 | .057 | .714 | | .025 | .057 | .656 | | .021 | .057 | .711 | |
| Mathematical competence | .022 | .062 | .727 | | .025 | .062 | .692 | | .028 | .062 | .656 | |
| Scientific literacy | -.172 | .065 | .008 | | **-.171** | .065 | .009 | | **-.170** | .065 | .009 | |
| Reading competence | -.022 | .061 | .717 | | -.020 | .061 | .743 | | -.022 | .061 | .723 | |
| Self-efficacy | **.198** | .053 | .000 | | **.197** | .053 | .000 | | **.200** | .053 | .000 | |
| GPA | -.111 | .091 | .223 | | -.131 | .092 | .154 | | -.127 | .092 | .167 | |
| Graduation in typical number of years of general education | .071 | .099 | .473 | | .060 | .099 | .545 | | .060 | .099 | .545 | |
| Participation in prevocational program before training | **-.257** | .124 | .038 | | **-.268** | .124 | .030 | | **-.264** | .124 | .033 | |
| Sex (female) | **.308** | .117 | .009 | | **.327** | .118 | .006 | | **.328** | .117 | .005 | |
| Migration background | .140 | .115 | .222 | | .134 | .114 | .240 | | .143 | .114 | .210 | |
| Low parental education | -.010 | .154 | .949 | | -.017 | .154 | .912 | | -.030 | .154 | .847 | |
| Company size < 10 | .076 | .109 | .483 | | .059 | .110 | .595 | | .057 | .110 | .603 | |
| Regional hiring challenges | .022 | .019 | .246 | | .022 | .019 | .239 | | .023 | .019 | .224 | |
| Constant term | **7.985** | .376 | .000 | | **8.030** | .380 | .000 | | **7.895** | .389 | .000 | |
| R² | .048 |  |  | | .051 |  |  | | .052 |  |  | |

*Note. n = 2,102; effects with an p<=.05 in bold type; to allow easier interpretation of the effect of overeducation, the reference category for educational achievement in M2a has been changed.*

*A3 Logistic regression models to explain PCT by educational match (Extended model)*

|  | M0b | | | M1b (undereducation) | | | M2b (overeducation) | | |
| --- | --- | --- | --- | --- | --- | --- | --- | --- | --- |
|  | AME | S.E. | p-value | AME | S.E. | p-value | AME | S.E. | p-value |
| No or low school-leaving qualification | **.068** | .015 | .000 | **.040** | .017 | .015 |  |  |  |
| Intermediate school-leaving qualification | Reference category |  |  |  |  |  | **-.040** | .015 | .010 |
| High school-leaving qualification | **-.054** | .013 | .000 | **-.043** | .015 | .003 | **-.061** | .018 | .001 |
| Share of low-qualified beginning trainees in occupation |  |  |  |  |  |  | **.038** | .014 | .007 |
| Share of at least intermediate-qualified beginning trainees in occupation |  |  |  | **-.023** | .009 | .011 |  |  |  |
| No or low school-leaving qualification * share of at least intermediate-qualified beginning trainees in occupation |  |  |  | -.016 | .015 | .279 |  |  |  |
| Intermediate school-leaving qualification * share of low-qualified beginning trainees in occupation |  |  |  |  |  |  | -.021 | .015 | .164 |
| High school-leaving qualification * Share of low-qualified beginning trainees in occupation |  |  |  |  |  |  | .014 | .023 | .555 |
| Polynomial Coefficients |  |  |  |  |  |  |  |  |  |
| No or low school-leaving qualification² |  |  |  | .000 (omitted) |  |  |  |  |  |
| Intermediate school-leaving qualification² |  |  |  |  |  |  | .000 (omitted) |  |  |
| High school-leaving qualification² |  |  |  |  |  |  | .000 (omitted) |  |  |
| Share of low-qualified beginning trainees in occupation² |  |  |  |  |  |  | -.005 | .007 | .445 |
| Share of at least intermediate-qualified beginning trainees in occupation² |  |  |  | -.006 | .007 | .364 |  |  |  |
| Control Variables |  |  |  |  |  |  |  |  |  |
| Entering preferred occupation | **-.042** | .011 | .000 | **-.042** | .011 | .000 | **-.042** | .011 | .000 |
| Realistic orientation | **-.031** | .008 | .000 | **-.034** | .008 | .000 | **-.034** | .008 | .000 |
| Investigative orientation | -.001 | .007 | .851 | -.001 | .007 | .911 | -.001 | .007 | .859 |
| Artistic orientation | **.030** | .006 | .000 | **.030** | .006 | .000 | **.030** | .006 | .000 |
| Social orientation | .009 | .007 | .161 | .010 | .007 | .147 | .010 | .007 | .159 |
| Enterprising orientation | **.016** | .007 | .028 | **.016** | .007 | .024 | **.016** | .007 | .023 |
| Conventional orientation | -.010 | .006 | .139 | -.009 | .006 | .167 | -.009 | .006 | .176 |
| Mathematical competence | **-.016** | .008 | .038 | -.013 | .008 | .091 | -.013 | .008 | .088 |
| Scientific literacy | .009 | .008 | .251 | .009 | .007 | .226 | .009 | .007 | .230 |
| Reading competence | **.021** | .008 | .009 | **.022** | .008 | .004 | **.022** | .008 | .004 |
| Self-efficacy | -.004 | .005 | .495 | -.004 | .005 | .455 | -.004 | .005 | .436 |
| GPA | **.049** | .010 | .000 | **.043** | .011 | .000 | **.043** | .011 | .000 |
| Graduation in typical number of years of general education | -.024 | .013 | .064 | -.024 | .013 | .070 | -.024 | .013 | .067 |
| Participation in prevocational program before training | .001 | .013 | .923 | .001 | .013 | .948 | .001 | .013 | .962 |
| Sex (female) | -.008 | .014 | .587 | -.003 | .014 | .853 | -.003 | .014 | .855 |
| Migration background | **.029** | .014 | .032 | **.030** | .014 | .029 | **.029** | .014 | .031 |
| Low parental education | -.017 | .017 | .313 | -.017 | .017 | .306 | -.017 | .017 | .319 |
| Company size < 10 | **.061** | .014 | .000 | **.053** | .014 | .000 | **.053** | .014 | .000 |
| Regional hiring challenges | .002 | .002 | .266 | .002 | .002 | .283 | .002 | .002 | .281 |
| Pseudo R² | .079 |  |  | .084 |  |  | .085 |  |  |

*Note. Reference group: Successful in first training year; n = 4,097; significance levels refer to AME coefficients; effects with an p<=.05 in bold type; to allow easier interpretation of the effect of overeducation, the reference category for educational achievement in M2b has been changed.*

*A4 Tobit regression models to explain training satisfaction by interest and skill congruence (Extended model)*

|  | M3a (+ interaction terms RIASEC) | | | M4a (+ interaction terms competences) | | | |  |
| --- | --- | --- | --- | --- | --- | --- | --- | --- |
|  | b | S.E. | p-value | | b | S.E. | p-value | |
| Realistic orientation | **.132** | .061 | .031 | | **.139** | .059 | .018 | |
| Investigative orientation | -.030 | .059 | .609 | | -.038 | .058 | .514 | |
| Artistic orientation | -.033 | .058 | .570 | | -.036 | .052 | .489 | |
| Social orientation | .045 | .058 | .439 | | .029 | .056 | .606 | |
| Enterprising orientation | -.032 | .058 | .585 | | -.027 | .057 | .634 | |
| Conventional orientation | .039 | .059 | .508 | | .039 | .059 | .512 | |
| Relevance of Realistic interests | -.033 | .183 | .858 | |  |  |  | |
| Relevance of Investigative interests | .003 | .090 | .978 | |  |  |  | |
| Relevance of Artistic interests | .030 | .085 | .721 | |  |  |  | |
| Relevance of Social interests | .003 | .116 | .981 | |  |  |  | |
| Relevance of Enterprising interests | -.103 | .152 | .504 | |  |  |  | |
| Relevance of Conventional interests | -.076 | .097 | .437 | |  |  |  | |
| Realistic orientation * relevance of Realistic interests | -.030 | .065 | .642 | |  |  |  | |
| Investigative orientation * relevance of Investigative interests | -.013 | .055 | .810 | |  |  |  | |
| Artistic orientation * relevance of Artistic interests | -.052 | .048 | .281 | |  |  |  | |
| Social orientation * relevance of Social interests | -.006 | .055 | .915 | |  |  |  | |
| Enterprising orientation * relevance of Enterprising interests | .014 | .049 | .779 | |  |  |  | |
| Conventional orientation * relevance of Conventional interests | -.054 | .051 | .293 | |  |  |  | |
| Mathematical competence | .023 | .062 | .710 | | .017 | .067 | .797 | |
| Scientific literacy | **-.176** | .065 | .007 | | **-.179** | .068 | .009 | |
| Reading competence | -.012 | .061 | .843 | | -.023 | .063 | .716 | |
| Importance of mathematical competence |  |  |  | | -.045 | .055 | .414 | |
| Importance of scientific literacy |  |  |  | | .046 | .062 | .457 | |
| Importance of reading competence |  |  |  | | -.113 | .063 | .072 | |
| Mathematical competence * importance of mathematical competence |  |  |  | | -.005 | .045 | .909 | |
| Scientific literacy * importance of scientific literacy |  |  |  | | .001 | .042 | .982 | |
| Reading competence * importance of reading competence |  |  |  | | -.061 | .051 | .235 | |
| Polynomial Coefficients |  |  |  | |  |  |  | |
| Realistic orientation² | .059 | .052 | .255 | |  |  |  | |
| Investigative orientation² | .007 | .043 | .864 | |  |  |  | |
| Artistic orientation² | -.007 | .045 | .879 | |  |  |  | |
| Social orientation² | -.058 | .047 | .221 | |  |  |  | |
| Enterprising orientation² | .002 | .038 | .948 | |  |  |  | |
| Conventional orientation² | .040 | .044 | .364 | |  |  |  | |
| Relevance of Realistic interests² | .060 | .078 | .438 | |  |  |  | |
| Relevance of Investigative interests² | .000 | .038 | .992 | |  |  |  | |
| Relevance of Artistic interests² | .002 | .026 | .933 | |  |  |  | |
| Relevance of Social interests² | .006 | .058 | .919 | |  |  |  | |
| Relevance of Enterprising interests² | .045 | .071 | .528 | |  |  |  | |
| Relevance of Conventional interests² | -.035 | .054 | .510 | |  |  |  | |
| Mathematical competence² |  |  |  | | .009 | .030 | .755 | |
| Scientific literacy² |  |  |  | | -.002 | .028 | .940 | |
| Reading competence² |  |  |  | | .037 | .032 | .243 | |
| Importance of mathematical competence² |  |  |  | | -.015 | .040 | .709 | |
| Importance of scientific literacy² |  |  |  | | .031 | .046 | .508 | |
| Importance of reading competence² |  |  |  | | .077 | .049 | .121 | |
| Control variables |  |  |  | |  |  |  | |
| Entering preferred occupation | .161 | .105 | .127 | | .138 | .105 | .189 | |
| No or low school-leaving qualification | -.204 | .124 | .100 | | -.228 | .126 | .072 | |
| High school-leaving qualification | **-.307** | .121 | .011 | | **-.284** | .119 | .018 | |
| Self-efficacy | **.200** | .055 | .001 | | **.197** | .054 | .000 | |
| GPA | -.111 | .092 | .228 | | -.120 | .091 | .185 | |
| Graduation in typical number of years of general education | .066 | .101 | .509 | | .068 | .099 | .496 | |
| Participation in prevocational program before training | -.240 | .126 | .057 | | -.238 | .124 | .055 | |
| Sex (female) | **.358** | .135 | .009 | | **.340** | .123 | .006 | |
| Migration background | .140 | .114 | .221 | | .127 | .115 | .270 | |
| Low parental education | .001 | .155 | .997 | | -.003 | .155 | .984 | |
| Company size < 10 | .066 | .111 | .552 | | .060 | .109 | .580 | |
| Regional hiring challenges | .022 | .019 | .253 | | .022 | .019 | .246 | |
| Constant term | **7.837** | .405 | .000 | | **7.882** | .383 | .000 | |
| R² | .060 |  |  | | .056 |  |  | |

*Note. n = 2,102; effects with an p<=.05 in bold type.*

*A5 Logistic regression models to explain PCT by interest and skill congruence (Extended model)*

|  | M3b (+ interaction terms RIASEC) | | | M4b (+ interaction terms competences) | | | |  |
| --- | --- | --- | --- | --- | --- | --- | --- | --- |
|  | AME | S.E. | p-value | | AME | S.E. | p-value | |
| Realistic orientation | **-.021** | .008 | .008 | | **-.032** | .008 | .000 | |
| Investigative orientation | .000 | .007 | .946 | | -.001 | .007 | .907 | |
| Artistic orientation | **.026** | .008 | .001 | | **.030** | .006 | .000 | |
| Social orientation | .006 | .007 | .402 | | .009 | .007 | .195 | |
| Enterprising orientation | **.016** | .007 | .024 | | **.015** | .007 | .035 | |
| Conventional orientation | -.008 | .007 | .208 | | -.008 | .006 | .208 | |
| Relevance of Realistic interests | .025 | .022 | .244 | |  |  |  | |
| Relevance of Investigative interests | **-.037** | .010 | .000 | |  |  |  | |
| Relevance of Artistic interests | **.028** | .010 | .005 | |  |  |  | |
| Relevance of Social interests | **.032** | .013 | .016 | |  |  |  | |
| Relevance of Enterprising interests | .005 | .017 | .780 | |  |  |  | |
| Relevance of Conventional interests | .005 | .011 | .650 | |  |  |  | |
| Realistic orientation * relevance of Realistic interests | **-.017** | .007 | .024 | |  |  |  | |
| Investigative orientation * relevance of Investigative interests | .002 | .006 | .711 | |  |  |  | |
| Artistic orientation * relevance of Artistic interests | -.010 | .005 | .053 | |  |  |  | |
| Social orientation * relevance of Social interests | -.001 | .006 | .918 | |  |  |  | |
| Enterprising orientation * relevance of Enterprising interests | -.001 | .006 | .836 | |  |  |  | |
| Conventional orientation * relevance of Conventional interests | -.003 | .006 | .613 | |  |  |  | |
| Mathematical competence | -.**017** | .007 | .021 | | **-.016** | .008 | .036 | |
| Scientific literacy | .011 | .008 | .150 | | .008 | .008 | .263 | |
| Reading competence | **.019** | .008 | .017 | | **.021** | .008 | .011 | |
| Importance of mathematical competence |  |  |  | | .007 | .006 | .287 | |
| Importance of scientific literacy |  |  |  | | **-.018** | .007 | .014 | |
| Importance of reading competence |  |  |  | | **-.022** | .007 | .001 | |
| Mathematical competence * importance of mathematical competence |  |  |  | | .000 | .007 | .992 | |
| Scientific literacy * importance of scientific literacy |  |  |  | | -.006 | .005 | .284 | |
| Reading competence * importance of reading competence |  |  |  | | -.006 | .005 | .307 | |
| Polynomial Coefficients |  |  |  | |  |  |  | |
| Realistic orientation² | .006 | .006 | .330 | |  |  |  | |
| Investigative orientation² | -.003 | .005 | .584 | |  |  |  | |
| Artistic orientation² | .001 | .005 | .895 | |  |  |  | |
| Social orientation² | .004 | .005 | .441 | |  |  |  | |
| Enterprising orientation² | -.001 | .004 | .761 | |  |  |  | |
| Conventional orientation² | -.001 | .004 | .798 | |  |  |  | |
| Relevance of Realistic interests² | .001 | .010 | .944 | |  |  |  | |
| Relevance of Investigative interests² | **.014** | .004 | .001 | |  |  |  | |
| Relevance of Artistic interests² | -.002 | .003 | .582 | |  |  |  | |
| Relevance of Social interests² | -.003 | .007 | .666 | |  |  |  | |
| Relevance of Enterprising interests² | -.008 | .009 | .390 | |  |  |  | |
| Relevance of Conventional interests² | -.002 | .006 | .689 | |  |  |  | |
| Mathematical competence² |  |  |  | | -.004 | .005 | .397 | |
| Scientific literacy² |  |  |  | | .000 | .004 | .982 | |
| Reading competence² |  |  |  | | -.003 | .004 | .470 | |
| Importance of mathematical competence² |  |  |  | | -.005 | .005 | .373 | |
| Importance of scientific literacy² |  |  |  | | **.017** | .005 | .002 | |
| Importance of reading competence² |  |  |  | | **.010** | .005 | .044 | |
| Control variables |  |  |  | |  |  |  | |
| Entering preferred occupation | **-.049** | .011 | .000 | | **-.044** | .011 | .000 | |
| No or low school-leaving qualification | **.054** | .014 | .000 | | **.061** | .015 | .000 | |
| High school-leaving qualification | **-.055** | .013 | .000 | | **-.050** | .014 | .000 | |
| Self-efficacy | -.005 | .005 | .373 | | -.003 | .006 | .555 | |
| GPA | **.046** | .010 | .000 | | **.047** | .011 | .000 | |
| Graduation in typical number of years of general education | -.021 | .013 | .097 | | -.024 | .013 | .058 | |
| Participation in prevocational program before training | -.003 | .013 | .801 | | .001 | .013 | .963 | |
| Sex (female) | -.030 | .015 | .048 | | -.005 | .015 | .742 | |
| Migration background | **.028** | .013 | .038 | | **.031** | .014 | .022 | |
| Low parental education | -.015 | .017 | .377 | | -.018 | .017 | .280 | |
| Company size < 10 | **.046** | .014 | .001 | | **.062** | .014 | .000 | |
| Regional hiring challenges | .002 | .002 | .261 | | .002 | .002 | .240 | |
| R² | .103 |  |  | | .086 |  |  | |

*Note. Reference group: Successful in first training year; n = 4,097; significance levels refer to AME coefficients; effects with an p<=.05 in bold type.*
